# Metabolic Disturbances Induced by Sleep Restriction as Potential Triggers for Alzheimer’s Disease

**DOI:** 10.3389/fnint.2021.722523

**Published:** 2021-09-03

**Authors:** Jesús Enrique García-Aviles, Rebeca Méndez-Hernández, Mara A. Guzmán-Ruiz, Miguel Cruz, Natalí N. Guerrero-Vargas, Javier Velázquez-Moctezuma, Gabriela Hurtado-Alvarado

**Affiliations:** ^1^Area of Neurosciences, Biology of Reproduction Department, Ciencias Biológicas y de la Salud, Universidad Autónoma Metropolitana, Unidad Iztapalapa, Mexico City, Mexico; ^2^Posgrado en Biología Experimental, Universidad Autónoma Metropolitana, Unidad Iztapalapa, Mexico City, Mexico; ^3^Instituto de Investigaciones Biomédicas, Universidad Nacional Autónoma de México, Ciudad Universitaria, Mexico City, Mexico; ^4^Departamento de Fisiología, Facultad de Medicina, Universidad Nacional Autónoma de México, Mexico City, Mexico; ^5^Instituto Mexicano del Seguro Social, Centro Médico Nacional Siglo XXI, Hospital de Especialidades, Unidad de Investigación Médica en Bioquímica, Mexico City, Mexico; ^6^Departamento de Anatomía, Facultad de Medicina, Universidad Nacional Autónoma de México, México City, Mexico

**Keywords:** sleep restriction, cognitive function, Alzheimer’s disease, metabolism, insulin resistance

## Abstract

Sleep has a major role in learning, memory consolidation, and metabolic function. Although it is known that sleep restriction increases the accumulation of amyloid β peptide (Aβ) and the risk to develop Alzheimer’s disease (AD), the mechanism behind these effects remains unknown. In this review, we discuss how chronic sleep restriction induces metabolic and cognitive impairments that could result in the development of AD in late life. Here, we integrate evidence regarding mechanisms whereby metabolic signaling becomes disturbed after short or chronic sleep restriction in the context of cognitive impairment, particularly in the accumulation of Aβ in the brain. We also discuss the role of the blood-brain barrier in sleep restriction with an emphasis on the transport of metabolic signals into the brain and Aβ clearance. This review presents the unexplored possibility that the alteration of peripheral metabolic signals induced by sleep restriction, especially insulin resistance, is responsible for cognitive deficit and, subsequently, implicated in AD development.

## Introduction

Sleep is a reversible physiological process characterized by the loss of consciousness, reduction in locomotor activity, and decreased response to external stimuli. It also includes important physiological changes that allow the organism to enter an energy-saving status (e.g., decreasing temperature, heart rate, and blood pressure) (Roffwarg et al., 1966; Glotzbach and Heller, 1976; Kräuchi et al., 2000).

The amount of sleep varies considerably from one person to another but on average most adults need about 7 to 8 h of sleep each night to feel well-rested (Hirshkowitz, 2004). Sleep is crucial for the regulation of endocrine functions (Alford et al., 1973; Spiegel et al., 1999; Leproult and Van Cauter, 2009), the immune response (Rico-Rosillo and Vega-Robledo, 2018), memory processing (Rasch and Born, 2013), brain plasticity (Abel et al., 2013), blood-brain barrier regulation (Gómez-González et al., 2013; He et al., 2014), and brain debris clearance (Xie et al., 2013).

Sleep consists of two distinct phases: rapid eye movement (REM) sleep and non(N)-REM sleep, which can be identified using polysomnography (Roffwarg et al., 1966; Hirshkowitz, 2004). NREM sleep is subdivided into three stages; Stages one and two are often considered “light sleep, ” whereas stage three, also called slow wave sleep (SWS), is the deepest stage. In humans, REM sleep and NREM sleep alternate throughout the sleep period (Roffwarg et al., 1966; Saper et al., 2001; Fuller et al., 2006; Irwin, 2015). Total sleep time and the length of each stage decrease throughout life. Particularly, the decrease of REM sleep and SWS is observed in aging (Roffwarg et al., 1966; Li et al., 2018). This fact is relevant in the context of cognitive functions because both REM sleep and SWS are essential for learning and memory consolidation (Ackermann and Rasch, 2014; Boyce et al., 2017).

The common approaches to investigate the physiological role of sleep are: total sleep deprivation (24 h or more without sleep), sleep fragmentation (multiple awaking during total sleep time), and sleep restriction, also known as partial sleep deprivation (sleeping less than the recommended time depending on the age) (Banks and Dinges, 2007; Reynolds and Banks, 2010).

Voluntary sleep restriction is a prevalent problem in modern society (Potter et al., 2016) that affects neurobehavioral and physiological functioning. It contributes to the development of a range of negative health outcomes, including cardiovascular diseases, type 2 diabetes, and neurodegenerative diseases (Kincheski et al., 2017; Chattu et al., 2018).

In this review, we discuss how sleep restriction is related to metabolic and cognitive impairment. Briefly, we propose that chronic sleep restriction may induce the most common neurodegenerative disease worldwide Alzheimer’s disease (AD), by impairing brain insulin signaling.

## The Cognitive Cost of Sleep Restriction

Cognitive functioning refers to multiple mental abilities, including learning and memory, and it naturally declines with age. The term “mild cognitive impairment” (MCI) has been used to describe minor cognitive problems that could predispose to the development of neuropathological or psychiatric conditions including AD, vascular dementia, and another types of dementia (Golomb et al., 2004). In the following sections, we restrict the use of the term “cognitive impairment” to refer to the memory deficit associated with sleep restriction, as studied in humans or animal models.

One meta-analysis showed that people who present lower cognitive performance (i.e., low working memory or attention) sleep less than 7 h per night (Mohlenhoff et al., 2018).

In adults, sleep shorter than 7 h in acute or prolonged periods compromises cognitive processes and alertness (Cousins and Fernández, 2019; Zhang et al., 2019). Chronic sleep restriction to either 6 h or 4 h per day for 14 days results in cumulative cognitive performance deficits, including deficits in working memory (Van Dongen et al., 2003; Rhea et al., 2017) This suggests that even relatively moderate sleep restriction has an important neurobiological “cost”. Extending the time of chronic sleep restriction to 6 weeks also impairs cognitive function, particularly spatial orientation and alertness; but these functions may be recovered after 2 nights of proper sleep (Van Dongen et al., 2003; Hennecke et al., 2021).

Worryingly, sleep restriction also affects children and adolescents. In children, sleep restriction increases sleepiness and inattentive behaviors (Fallone et al., 2001). Also, a single night of sleep restriction (>5 h in bed) induced a deficit in verbal creativity and abstract thinking (Randazzo et al., 1998). Adolescents commonly get less than the recommended 8 to 10 h of sleep per night (Owens et al., 2014), which has been associated with a deficit in working memory (Short and Chee, 2019) and executive functions (Lo et al., 2016).

Short sleeping time is often associated with impaired performance in memory tasks (Mantua and Simonelli, 2019); in addition, the relationship between sleep, cognitive deficit, and the prevalence of AD has been confirmed by a meta-analysis (Bubu et al., 2017). A study of nearly 8,000 people found that those who consistently got 6 h of sleep or less per night during 25 years from their 50 s to 60 s were 30 percent more likely to develop dementia later in life, compared with those who slept 7 h per night (Sabia et al., 2021).

β-amyloid peptide (Aβ) aggregation is present in preclinical stages of AD; the excitotoxic and neuroinflammatory properties of Aβ seem to play an important role in the induction of neurodegeneration (Tatarnikova et al., 2015). Another characteristic of AD is the disturbance of the cytoskeleton in nerve cells, which is associated with the hyperphosphorylation of Tau-protein (Tatarnikova et al., 2015).

Sleep restriction worsens the memory impairment and Aβ deposition in a mouse model of AD (Rothman et al., 2013); it can also induce cognitive impairment and Aβ accumulation in animal models without a genetic predisposition to develop AD. Sleep restriction increases the levels of Aβ in the hippocampus (Brice et al., 2020), the expression of Aβ_42_ and Aβ_1–40_, and the levels of the enzyme β-secretase that cleaves the Amyloid Precursor Protein (APP) for the synthesis of Aβ (Chen et al., 2017). Recently, studies performed in young mice demonstrated that chronic sleep restriction for 12 months induces hippocampal neurodegeneration along with spatial memory deficits, neuroinflammation, gliosis, and an increase in Aβ_42_ and hyperphosphorylated tau protein (Owen et al., 2021). Chronic sleep restriction in C57BL/6J mice decreases the number of neurons and volume of the CA1 region of the hippocampus, resulting in lower performance in the conditioning test of place. In addition, lower performance in this test correlated with an increased concentration of Aβ (Owen et al., 2021). This evidence reinforces the idea that Aβ accumulation due to sleep restriction can induce cognitive impairment and neurodegeneration without a genetic predisposition to develop AD.

Several authors have discussed the association between sleep and the pathogenesis of AD *via* Aβ and other factors like Tau-protein; and some have proposed sleep interventions to reduce AD symptoms (Cordone et al., 2019, 2021; Wang and Holtzman, 2020; Özcan et al., 2020).

Age plays an important role in cognition since aging is characterized by a decrease in REM sleep, an increase in NREM sleep and, frequently, sleep fragmentation, which is related to cognitive decline (Berkley, 2021).

A clinical study in older participants without dementia (>60 years) reported that participants that slept less than 6 h had significantly higher levels of circulating Aβ42 oligomer than those that slept for more than 7 h (Liu et al., 2021).

Furthermore, another study using positron emission tomography (PET), performed in 22 healthy participants (age 22 to 72 years old), revealed that only one night of sleep loss increased the levels of Aβ (Shokri-Kojori et al., 2018). Interestingly, the CSF Aβ_42_ levels of the participants between the ages of 40 and 60 years old were the highest of all (Ooms et al., 2014), suggesting that sleep loss interferes with the clearance of Aβ (Mendelsohn and Larrick, 2013).

Inflammation is probably one of the mechanisms by which sleep restriction alters brain physiology. During sleep restriction, there is an increase in proinflammatory molecules in the hippocampus (Zielinski et al., 2014; Kincheski et al., 2017), although the origin of these molecules is not well understood. Access of circulating molecules into the hippocampus is limited by the BBB, but several circulating inflammatory molecules, and metabolic signals can cross it. In addition, metabolic signals such as insulin participate in the modulation of synaptic plasticity in the hippocampus (Banks, 2006; Peineau et al., 2018; Lyra e Silva et al., 2021).

Unlike neuroinflammation and neurodegeneration, which are detected after prolonged sleep restriction, systemic changes in metabolic and inflammatory mediators appear after a single night without sleep (Zielinski et al., 2014). Thus, chronic sleep loss could induce a sustained impairment in the signaling of these mediators, contributing to the development of cognitive detriments, and chronically increasing the risk to develop AD.

## Sleep Restriction Alters Metabolic Regulation: The Link for Cognitive Impairment

Chronic sleep restriction induces metabolic alterations that lead to metabolic disorders, which in turn are known risk factors for developing AD (Ott et al., 1999; Whitmer et al., 2005, 2008). Acute and chronic sleep loss induces glucose intolerance and insulin resistance; decrease insulin-like growth factor (IGF)-1 and adiponectin levels (Van Leeuwen et al., 2010; Broussard et al., 2015, 2016); and induce dyslipidemia and systemic low-grade inflammation (Gangwisch et al., 2010; Broussard et al., 2015) in non-obese subjects (humans and animal models).

How could the metabolic impairment caused by sleep restriction be the origin of cognitive disturbances? In the next section, we will discuss the metabolic signals associated with sleep restriction that have a direct effect on brain function and cognitive performance.

### Insulin: the Main Suspect

Insulin is a hormone secreted by the β cells of the pancreas (Thevis et al., 2009) that has a key role in maintaining normal blood glucose levels by facilitating cellular glucose uptake (Dimitriadis et al., 2011). Insulin resistance occurs when insulin-dependent tissues require increased concentrations of this hormone to achieve the biological effects (Wilcox, 2005).

Sleep loss reduces insulin sensitivity in healthy individuals (Spiegel et al., 2005; Buxton et al., 2010; McNeil et al., 2013; for review see Koren et al., 2015). In humans, a single night of less than 4 h of sleep significantly raises blood glucose levels and reduces insulin sensitivity (Donga et al., 2010). The same has been observed in rats subjected to 4 h of sleep restriction at the beginning or in the middle of the rest period (Jha et al., 2016). On the contrary, recovering normal sleep time is associated with an improvement in insulin sensitivity (Simon et al., 2019). This contributes to the proposal that avoiding sleep loss may help to prevent the development of metabolic syndrome (Leproult and Van Cauter, 2009).

Insulin is necessary for memory and learning (Zhao et al., 2004). Insulin receptors are expressed in brain areas such as the olfactory bulb, hypothalamus, and hippocampus (Kleinridders et al., 2014; Soto et al., 2019). Insulin crosses the BBB in the hippocampus (Spinelli et al., 2019) and regulates the recruitment of *N*-methyl-D-aspartate (NMDA) receptors in excitatory synapses (Skeberdis et al., 2001), contributing to hippocampal long-term potentiation (LTP) (Van Der Heide et al., 2005). At the same time, insulin is related to the regulation of α-amino-3-hydroxy-5-methyl-4-isoxazolepropionic acid (AMPA) receptors by modulating the endocytosis of these receptors through phosphatidylinositol 3-kinase/protein kinase C (PI3K-PKC; Huang et al., 2004).

Brain insulin resistance can be defined as a failure of brain cells to respond to insulin (Arnold et al., 2018). Memory and learning are impaired in animal models of insulin resistance (Park et al., 2013; Grünblatt et al., 2015; Pratchayasakul et al., 2015), but some studies indicate that intracerebral administration of insulin is able to restore cognitive function (Haj-ali et al., 2009). These studies indicate that insulin transport from the blood into the brain is crucial for cognition through neuronal signaling.

This hypothesis is supported by the fact that insulin receptor (IR), and IGF-1 receptor (IGF1R) specific knock-outs in the hippocampus and in the amygdala not only decrease the expression of AMPA receptors, but also develop into glucose intolerance, anxiety-like behaviors and impaired memory recognition, and spatial memory (Soto et al., 2019).

In AD patients, the activation of the IR is reduced, mainly in the hippocampus and hypothalamus, because of the phosphorylation of serine-residues in the IR substrate (IRS). This phosphorylation prevents the translation of the insulin signal upon binding to the IR (Steen et al., 2005). Furthermore, *ex vivo* insulin stimulation activated the canonical signaling pathways significantly less in post-mortem brain sections of AD patients as compared to those of healthy subjects, suggesting the presence of central insulin resistance in AD (Talbot et al., 2012).

Also, the inoculation of a viral vector containing short hairpin RNA (ShRNA) against human and rat IRS1 into the dorsal hippocampus of Wistar rats decreased their performance on the T-maze cognitive test and the novel object recognition test, in the absence of pro-inflammatory cytokines (Sánchez-Sarasúa et al., 2021).

These results suggest that brain insulin resistance might be a plausible mechanism that increases the incidence of AD after sleep restriction. Up until now, there is no evidence indicating if sleep restriction induces central insulin resistance; therefore, more studies are needed to explore this relationship. These studies should aim to test whether sleep restriction changes brain IR, modifies insulin transport through the BBB, or promotes any other change in insulin signaling.

### Glucose, Hyperglycemia, and Advanced Glycation End Products

Chronic hyperglycemia associated with insulin resistance could influence cognitive performance. Hyperglycemia is associated with deterioration in mood and cognitive function in patients with type 2 diabetes (Greenwood et al., 2003; Sommerfield et al., 2004).

Chronic hyperglycemia leads to the accelerated formation of advanced glycation end products (AGEs), commonly observed in obesity, diabetes, and AD (Cai et al., 2016; Pugazhenthi et al., 2017). AGEs are a very large group of molecules formed from non-enzymatic glycation reactions that affect the structure and function of proteins, amino acids, and nucleic acids (Singh et al., 2001). Under inflammatory conditions such as acute sepsis, the interaction between AGEs, and their receptors (RAGE) in the brain results in the activation of pro-inflammatory genes in the blood, hippocampus, and prefrontal cortex. These changes are associated with neuroinflammation, increased Aβ immunodetection, and enhanced Tau phosphorylation in the hippocampus. In this model, blocking RAGE inhibits neuroinflammation and neurodegeneration markers (Gasparotto et al., 2018). Similar findings indicating that RAGE antagonist or RAGE knockout mice fail to present spatial memory deficits suggest that AGES play a role in cognitive impairment (Cai et al., 2016; Pugazhenthi et al., 2017; Momeni et al., 2021).

In the brain, exposure to high glucose levels for 24 h increases the expression of ionic cotransporters associated with edema formation in rodents (Klug et al., 2021). It also alters brain endothelial cell function and decreases GLUT1 expression, which results in a decrease of glucose uptake into the brain (for a review see Leão et al., 2020). Further, Aβ accumulation is associated with the decrease in membrane expression of the glucose transporter GLUT1 (Winkler et al., 2015), and microvascular endothelial cells are more susceptible to Aβ toxicity under hyperglycemic conditions, suggesting that hyperglycemia is also a risk factor for vascular damage associated with AD (Carvalho et al., 2014). Indeed, AD and type 2 diabetes patients with hyperglycemia have lower glucose uptake by the brain (Hendrix et al., 2021). This suggests that the brain’s glucose supply is altered during neurodegenerative events and that other mechanisms such as the action of AGEs could be involved in the cognitive impairment observed in these patients.

These mechanisms could also be involved in sleep restriction-associated cognitive impairment, because: (1) isolated brain endothelial cells from sleep-restricted mice have lower levels of GLUT1 and decreased 2-deoxy-glucose uptake in the brain (He et al., 2014); and (2) sleep restriction increases the plasmatic soluble RAGE in rats (Liu et al., 2020).

### Low-Grade Inflammation

Sleep loss induces a chronic low-grade inflammatory state similar to those observed under obese conditions: it involves a subtle but sustained increase of interleukin 6 (IL-6), tumor necrosis factor (TNF)-α, interleukin 1 (IL-1), interleukin 17A (IL-17A), and C-reactive protein (CRP) (for review (Hurtado-Alvarado et al., 2013; Irwin et al., 2016). Increased IL-6 and TNF-α plasma levels have been associated with glucose intolerance and a reduction of IRS1 in the muscle of sleep-restricted rats (Venancio and Suchecki, 2015). Low-grade inflammation is related to the incidence of AD (Tao et al., 2018), diabetes, and cardiovascular diseases (Vinuesa et al., 2021). In a metabolic context, some pro-inflammatory cytokines such as TNF-α influence insulin sensitivity by activating pathways such as IKK, JNK, and MAPK in the adipose tissue and muscle of obese animals (Hotamisligil, 2006; Chan et al., 2019).

Proinflammatory cytokines in the brain increase after one day of sleep restriction (Zielinski et al., 2014). Those inflammatory factors correlate with Aβ_42_ deposition and a higher expression of RAGE in the hippocampus and cortex (Liu et al., 2020). IL-1β and TNF-α in the brain also correlate with the increase in β-site APP-cleaving enzyme 1 (BACE1) which is a key molecule that facilitates Aβ accumulation (Liu et al., 2020). Particularly, the high expression of TNF-α in the hippocampus of sleep-restricted animals decreases synaptic connections, as well as performance in the novel object recognition test (Kincheski et al., 2017).

In AD mouse models, TNF-α signaling in hippocampal neurons mediates synapse loss and memory impairment by disrupting insulin signaling (Lourenco et al., 2013). Moreover, IL-6 has been proposed as a key signal that links memory impairment and metabolic dysfunction in AD (Lyra e Silva et al., 2021).

Peripheral signals secreted after sleep restriction are necessary to initiate hippocampal inflammation since BALB/c mice, which have a predominant Th2 activation and fail to produce systemic proinflammatory cytokines after sleep restriction, do not overexpress neuroinflammatory markers in the hippocampus (Hurtado-Alvarado et al., 2018). Thus, the proinflammatory cytokines induced by sleep restriction (e.g., TNF-α and IL-6) could contribute to the development of cognitive impairment and participate in the development of neuroinflammation in the regions involved in memory and learning, probably by modifying insulin sensitivity in the brain.

### Other Metabolic Signals Altered by Sleep Restriction

#### Free Fatty Acids

The sleeping period is accompanied by several hours of fasting, in which the main energy substrate is provided by free fatty acids (FFA) that are released into the circulation from the white adipose tissue (Shostak et al., 2013; Kumar Jha et al., 2015). Under sleep restriction, energy demands are increased due to the waking state of the individual (St-Onge, 2017); if food is not available, these demands could be met by an increased glucose production from the liver, or, alternatively, by increased FFA release and oxidation. Because insulin resistance occurs shortly after sleep deprivation, it is unlikely that glucose would serve as a fuel source in this condition. Indeed, endogenous glucose production does not increase during sleep deprivation (Knutson, 2007), thus, energy requirements must be met with an increase in FFA oxidation. One study found that four consecutive nights of sleep loss in healthy men are associated with an increase in circulating FFA (Broussard et al., 2015). Also, prolonged REM sleep restriction (21 days) in rats produces a substantial decrease in retroperitoneal adipose tissue mass (Venancio and Suchecki, 2015), indicating that lipid mobilization takes place under this condition.

In addition, sleep loss is also associated with an increase in food consumption and preference for highly energetic lipid-rich food (Knutson, 2007; Hogenkamp et al., 2013; Briançon-Marjollet et al., 2015). In this sense, FFA coming either from triglyceride storage or from a high-energy meal could contribute to elevated FFA levels during sleep restriction. In addition, the increase in FFA levels after sleep restriction correlates with insulin resistance (Broussard et al., 2015). In accordance with this, some authors have hypothesized that insulin resistance could be caused by high levels of FFA, which stimulate the production of cytokines in the periphery but also in the brain (Roden et al., 1996; Sears and Perry, 2015). FFA can bind to toll-like receptors in the brain, contributing to an increased inflammatory status (Könner and Brüning, 2011). These observations suggest that increased FFA levels after sleep deprivation could be related to insulin resistance and neuroinflammation, thus providing a possible explanation for how this cascade of events is initiated. More research is still necessary to evaluate if FFA indeed contributes to insulin resistance and the subsequent cognitive impairment observed after sleep restriction, as well as to dissect the contribution of each type of FFA (short chain/long chain, saturated/unsaturated) in these processes.

#### Insulin-Like Growth Factor-1

IGF-1 is part of the IGF signaling system (also composed of insulin, and insulin receptors) produced mainly in the liver (70%), and its receptor IGF-1R is found predominantly in the hippocampus (Adem et al., 1989; Doré et al., 1997). A single night of sleep loss caused a decrease in circulating IGF-1 levels in humans (Chennaoui et al., 2014) and rats (Everson et al., 2012). Interestingly, the metabolic disturbances induced by sleep loss in rats, such as hyperglycemia and elevated blood pressure can be prevented by a daily administration of systemic IGF-1 (Chen et al., 2014).

Several studies demonstrate that IGF-1 has a key role in synaptic excitability (Xing et al., 2007) and hippocampal neuron polarity (Sosa et al., 2006). It also promotes LTP in the prefrontal cortex and hippocampus, and a decrease in IGF-1 is considered to be an important marker in age-related memory decline (Lynch et al., 2001; Burgdorf et al., 2015).

Defective insulin and IGF-1 signaling in the brain are associated with neurological disorders such as AD (for review see Ferreira, 2021). Moreover, IGF-1/ IGF-1R signaling in the hippocampus is altered in a rat model of vascular dementia; its downregulation contributes to impaired learning and memory (Gong et al., 2012). More evidence is needed to elucidate the role of IGF-1 in the brain after sleep restriction.

#### Adiponectin

Adiponectin is an adipokine secreted by the adipose tissue in inverse proportion to the amount of fat. Only a handful of studies have described the effects of sleep restriction on adiponectin levels in humans, and the results are controversial. Kotani et al. (2007) found that the amount of systemic adiponectin was negatively correlated with hours of sleep in a study of 109 healthy men. Conversely, a study conducted on Caucasian and African American men and women who were only allowed 4 h of bedtime for 5 consecutive days found that only Caucasian women showed decreased levels of adiponectin (Simpson et al., 2010).

The effect of sleep restriction on adiponectin levels in children and adolescents has also been studied. Hitze et al. (2009) found that sleeping less than 10 h for children or 9 for adolescents correlated with lower levels of adiponectin. As far as we know, there is no evidence of adiponectin changes associated with sleep restriction in animal models. In fact, only one study in rats subjected to sleep deprivation for 96 h measured adiponectin levels and did not find changes (Rosa Neto et al., 2010).

Despite the lack of conclusive evidence, adiponectin is an important candidate for determining the molecules involved in the detrimental effects of sleep restriction in cognition since: (1) adiponectin increases insulin sensitivity (Fang and Judd, 2018); (2) it is considered an anti-inflammatory and protective agent of the vasculature (including BBB; Fang and Judd, 2018); (3) adiponectin receptors are expressed in the central nervous system (CNS; Bloemer et al., 2018); and (4) adiponectin knockout mice perform poorly on the new object recognition test and exhibit LTP deficiency (Bloemer et al., 2019). Further, in a rat model in which olfactory memory impairment and olfactory damage were caused by intracerebroventricular administration of Aβ_41–42_, intracerebroventricular (icv) adiponectin prevented olfactory memory impairments (Guzmán-Ruiz et al., 2021), which are common early symptoms in patients with MCI and AD.

The study of the central and peripheral alterations of this adipokine in animal models of sleep restriction could provide a potential target to improve insulin resistance and the associated cognitive impairments.

#### Leptin

Sleep restriction causes a decrease in systemic leptin levels (Spiegel et al., 2004; Taheri et al., 2004). However, the effect of decreased sleep time on systemic leptin levels in humans is controversial. Some reports indicate leptin increases after sleep restriction while systemic meta-analysis indicates that the evidence supporting leptin changes is not strong enough (Reynolds et al., 2012; Zhu et al., 2019).

Still, the role of leptin, an adipokine involved in energy, is worthy to be considered. In rats, sleep restriction decreases leptin levels concomitant to increasing proinflammatory cytokines and decreasing glucose tolerance (Leproult and Van Cauter, 2009; Venancio and Suchecki, 2015; Chen et al., 2017).

Leptin receptors are expressed in different brain regions, including the hippocampus (Håkansson et al., 1998), where leptin can promote spatial learning (Drel et al., 2006) probably *via* IL-1β receptors (Erion et al., 2014). Mice that do not express the leptin receptor (db/db) exhibit cognitive deficits (Dinel et al., 2011), and obese rodents, which are insensitive to leptin, also show cognitive deficits and have deteriorated LTP (Li et al., 2002; Winocur et al., 2005). The effects of leptin deficiency on cognitive function have been attributed to inflammatory events in obese models, but the role of leptin and the leptin receptor has also been studied in an AD model (Pratap and Holsinger, 2020).

Circulating molecules require access to the brain to display their central function. In this way, the BBB serves as an interface between peripheral signals and the neural environment that is dramatically altered under sleep restriction (Gómez-González et al., 2013; He et al., 2014; Hurtado-Alvarado et al., 2016a, 2018). Moreover, under pathological conditions such as diabetes and AD, there is a reduction in the access of metabolic signals into the brain concomitant to neuroinflammation and blood-brain barrier disruption (Banks, 2006, 2020; Rhea et al., 2017; Rhea and Banks, 2021). Hence, the role of the blood-brain barrier should be considered in the mechanisms by which sleep restriction alters cognitive function.

## The Role of the Blood-Brain Barrier in Cognitive Impairment Associated With Sleep Restriction

The optimal function of the BBB is essential for brain function. The BBB limits non-specific transport between endothelial cells by tight junctions and low level of pinocytosis limiting paracellular diffusion of hydrophilic compounds (for review see Gómez-González et al., 2012; Daneman and Prat, 2015; Profaci et al., 2020). The general components of the BBB are microvascular brain endothelial cells, pericytes, astrocytes, and capillary adjacent microglia. The expression of transporters and receptors in brain endothelial cells is responsible for the active transport of circulating nutrients into the brain (e.g., glucose by GLUT1) or the efflux of metabolites from the brain to the circulation such as lipoprotein receptor-related protein 1 (LRP1) (for review see Gómez-González et al., 2012; Daneman and Prat, 2015; Profaci et al., 2020).

Sleep-induced changes in the BBB are related to neuroinflammation, oxidative stress, excitotoxicity, and subsequent neuronal loss (Engelhardt and Sorokin, 2009; Gómez-González et al., 2012). Sleep restriction changes BBB morphology and function. In rats, 10 days of sleep restriction (4 h of sleep per day) increases the BBB permeability to Evans blue, sodium fluorescein, and dextrans (10 and 70 kDa) by the disruption of tight junctions and the increase in pinocytosis (Gómez-González et al., 2013; Hurtado-Alvarado et al., 2016b, 2018). Also, mice that were restricted for 6 days (sleep period 6 h per day) increased their BBB permeability to sodium fluorescein by tight junction disentanglement (He et al., 2014).

Interestingly, the hippocampal BBB permeability of sleep-restricted rats remains higher after 2 h of sleep recovery, unlike other brain areas such as the cortex and basal nuclei that have a complete restoration of basal BBB in this period (Gómez-González et al., 2013; Hurtado-Alvarado et al., 2017). However, 24 h of sleep recovery after sleep restriction is enough to restore the normal permeability of the BBB (He et al., 2014).

Impaired BBB function also affects other components of the neurovascular unit. Sleep restriction induces overexpression of markers classically associated with gliosis such as iba-1 for microglia and glial fibrillar acidic protein (GFAP) for astrocytes. It also increases the expression of the A_2A_ receptor and matrix metalloproteinase (MMP)-9 and induces detachment of pericytes from endothelial cells (Hurtado-Alvarado et al., 2016b; Medina-Flores et al., 2020).

Sleep restriction increases the Aβ deposition in the cortex and hippocampus of sleep-restricted animals (Zhao et al., 2019; Owen et al., 2021). A proposed mechanism that leads to Aβ accumulation is the decrease in LRP1 levels in the brain (Zhao et al., 2019). LRP1 is a major endothelial surface receptor that mediates clearance of Aβ across the BBB so that it may circulate towards the liver and kidneys for systemic clearance (for review see Cockerill et al., 2018). Sleep restriction also increases RAGE in the hippocampus and prefrontal cortex (Zhao et al., 2019). In normal conditions, RAGEs are responsible for the transport of Aβ from the circulation into the brain. Thus, increasing their expression during sleep restriction could further contribute to Aβ deposition in the brain (Deane et al., 2003).

Even though Aβ clearance mechanisms in the BBB remain to be explored under sleep restriction, it is known that Aβ levels in the brain fluctuate along the day, with lower levels during the resting period (Roh et al., 2012; Lucey et al., 2017). This agrees with the evidence that shows that Aβ is largely cleared *via* the brain’s glymphatic system (Xie et al., 2013).

## Hypothetical Mechanism of AD Development Induced by Chronic Sleep Restriction

AD is considered by some authors as a CNS pathology caused by the dysfunction of the BBB. This is based on the hypothesis that Aβ decomposition in the CNS initiates a molecular cascade that causes neurodegeneration (Cockerill et al., 2018). Further, some authors have published reviews that integrate the role of insulin in Aβ transport in the brain and the consequences this may have on AD development (Vandal et al., 2015; Mullins et al., 2017; Rhea et al., 2020; Alves et al., 2021; Rhea and Banks, 2021). Based on the evidence presented, we propose that chronic sleep restriction induces peripheral low-grade inflammation and insulin resistance leading to hyperglycemia which together alter the BBB function (Figure 2). The BBB deterioration associated with sleep restriction can allow the entrance of exogenous molecules such as albumin, dyes, or toxic molecules (i.e., glutamate, urea) but probably impair the entrance of glucose *via* GLUT1 reduction and central insulin resistance. Since there is a tight relationship between LRP1 levels and IR signaling (Gali et al., 2019), it is possible that, in sleep-restricted rats, the decrease in LRP1 (Zhao et al., 2019) is associated with altered IR signaling, resulting in insulin resistance. Insulin resistance could be involved in the upregulation of RAGE, which would decrease the efflux of Aβ from the brain into the blood. Despite the lack of evidence regarding the relationship between insulin and RAGE in the BBB, mice administrated with streptozotocin (that causes insulin deficiency and type 1 diabetes) have an upregulation of RAGE which would contribute to Aβ deposition in this model (Liu et al., 2009). The decrease in circulating molecules such as adiponectin and IGF-1 during sleep restriction could contribute to the establishment of brain insulin resistance while FFAs could also worsen systemic insulin resistance.

**Figure 1:**
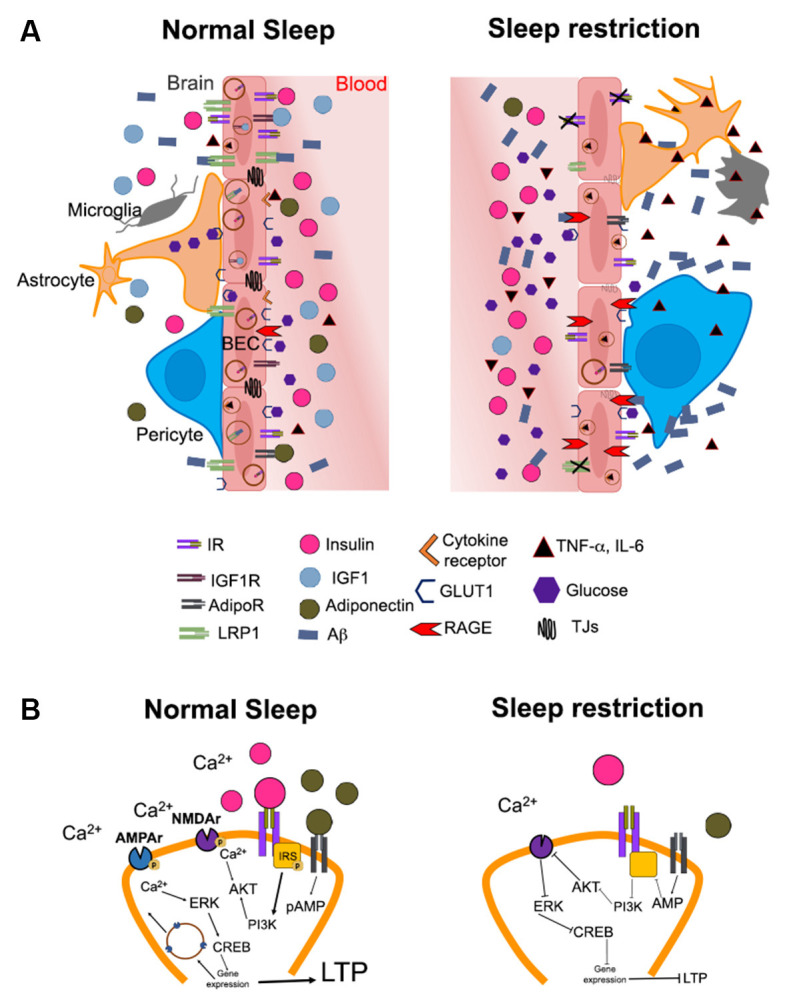
Hypothetical mechanisms by which altered brain insulin signaling induced by sleep restriction may promote the development of Alzheimer’s disease. **(A)** The illustration shows the possible changes in the access of metabolic signals into the brain (e.g., hippocampus) during normal sleep and sleep restriction. Substances in the periphery are limited in their passage to the brain due to the blood-brain barrier (BBB). The BBB is constituted by brain endothelial cells, pericytes, astrocytes, microglia, and neurons. In normal sleeping conditions (left), metabolic signals such as IGF-1 (blue circles), adiponectin (green circles), glucose (purple hexagons), and insulin (pink circles) circulate in the appropriate concentrations through blood vessels and may bind to their receptors in the brain. The functions of insulin signaling include the modulation of low-density lipoprotein receptor-related protein 1 (LRP1) expression, which mediates amyloid beta (Aβ, blue rectangles) efflux into the bloodstream. IGF-1 and adiponectin bind to their receptors on endothelial cells, possibly increasing insulin sensitivity and modulating the expression of LRP1 receptors. In addition, glucose enters the brain through glucose transporter 1 (GLUT1) for proper brain functioning. Conversely, sleep restriction (right) increases peripheral levels of pro-inflammatory cytokines such as TNF-α and IL-6 (black triangles). The components of the BBB show morphological changes, probably due to an increase in pro-inflammatory cytokines and a decrease in adiponectin and IGF-1. Pericytes detach from endothelial cells, in addition, both astrocytes and microglia change to an “active” morphology due to inflammation. There is also a decrease in the expression of LRP1 due to the impairment in insulin signaling and the decrease of IGF-1 and adiponectin. In this way, the efflux of Aβ from the brain to the blood is decreased, while the expression of receptors for advanced glycation end products (RAGE), which can recapture circulating AB, is increased. The decrease in glucose uptake mediated by GLUT1 during sleep restriction could further contribute to hyperglycemia and the formation of advanced glycation end products (AGEs). In addition, the increase or maintenance of peripheral insulin levels (peripheral insulin resistance) is associated with a decrease in the entrance of insulin into the brain (central insulin resistance), probably due to decreased expression of the insulin receptor (IR). AdipoR and IGF-1 could also decrease in these conditions, although this is not yet fully understood. **(B)** In normal conditions (left), the binding of the insulin receptor (IR) with its ligand and/or adiponectin with its receptor (AdipoR) causes phosphorylation of the insulin receptor substrate (IRS, yellow square) triggering a signaling cascade that involves the activation of phosphatidylinositol-3 kinase (PI3K) and protein kinase B (AKT). This promotes phosphorylation of the N-methyl-D-aspartate (NMDA) receptor (purple figure) and Ca^2+^ influx into the neuron. The increase in intracellular Ca^2+^ promotes the activation of cAMP response element-binding protein (CREB), which functions as a transcription factor for genes that promote the mobilization of α-amino-3-hydroxy-5-methyl-4-isoxazolepropionic acid (AMPA) receptors (blue) to the cell membrane. These events promote long-term potentiation (LTP). On the contrary, during sleep restriction (right), the decrease in insulin in the brain parenchyma combined with the decrease in the concentration of adiponectin, causes a decrease in the phosphorylation of the insulin receptor substrate; therefore, the activation of this signaling pathway is diminished. This decreases AMPA receptors and prevents activation of NMDAs, ultimately producing a decrease in the expression of LTP-related genes.

**Figure 2:**
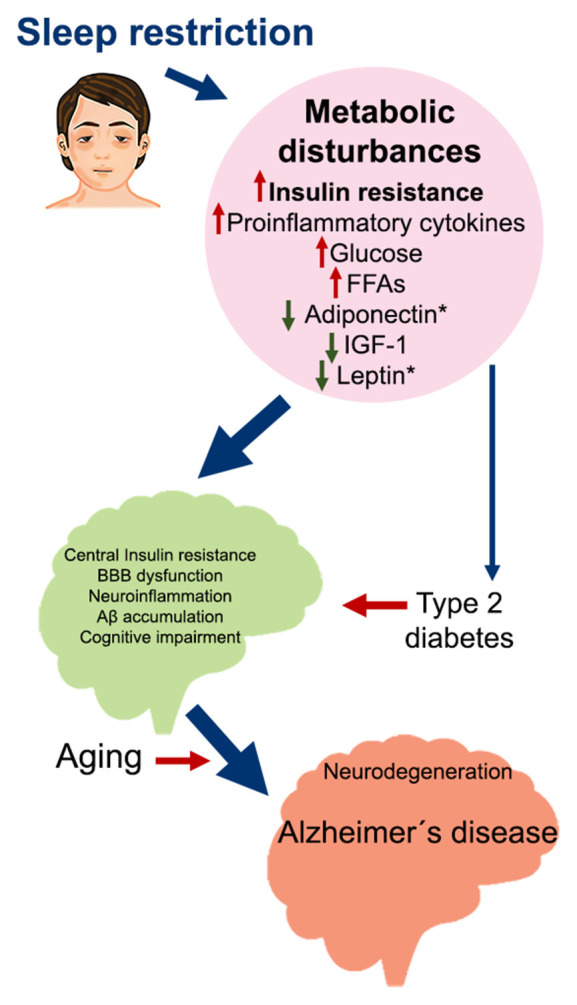
Metabolic impairment induced by sleep restriction in the development of Alzheimer’s disease. Proposed sequence of events that may trigger Alzheimer’s disease as a consequence of chronic sleep restriction. Sleep restriction induces several metabolic disturbances in the periphery, including insulin resistance; increased proinflammatory cytokines, glucose, and FFA; and decreased adiponectin, IGF-1, and leptin. These changes can promote several alterations in the brain, such as central insulin resistance, BBB dysfunction, neuroinflammation, Aβ accumulation, and cognitive impairment. At the same time, persistent metabolic disturbances may develop into Type 2 diabetes, which is also associated with the same brain alterations. On top of this, aging broadens the risk for developing Alzheimer’s disease and probably other neurodegenerative diseases. Abbreviations: FFA, free fatty acids; IGF-1, insulin-like growth factor 1; BBB, blood-brain barrier.

Insulin resistance induced by sleep restriction could also contribute to Aβ accumulation in the brain by the regulation of LRP1 and RAGE function (Figure 1), driving neuroinflammation, neurodegeneration, and cognitive impairment, all factors that contribute to AD development (Figure 2).

In humans, other conditions such as obesity (Simon et al., 2021), circadian disruption (Bolsius et al., 2021), changes in gut microbiota (Ekundayo et al., 2021), and sleep disturbances like obstructive apnea (Siachpazidou et al., 2020) coexist with sleep restriction, which makes it difficult to separate the effects of sleep restriction from the others. In addition, these conditions have a direct relationship with sleep quality, which further promotes the coexistence of these factors. In this sense, bad habits could establish a vicious cycle between metabolic impairments, cognitive deficit, and sleep deficiency, potentially increasing the risk of AD development.

## Further Considerations and Concluding Remarks

Chronic sleep loss because of voluntary bedtime restriction is an endemic condition in modern society. Moreover, sleep restriction is a health problem observed since childhood. The fact that children and adolescents get less hours of sleep than recommended and that this has an impact on metabolism is worrying in an obese-prone society. As well as obesity prevention, interventions associated with sleep quality should be proposed as a strategy to reduce AD symptoms (Cordone et al., 2019, 2021).

Special interest has been placed in sleep hygiene to improve the quality of sleep. In this way, it is suggested to avoid the use of psychoactive substances at night (Cordone et al., 2021) such as caffeine (Roehrs and Roth, 2008), energy drinks (Sampasa-Kanyinga et al., 2018), and smoking (McNamara et al., 2014). Conversely, there are various drugs that are used to treat sleep deficiency in AD patients. One of them is melatonin, which improves the amount of sleep in these patients (Xu et al., 2015). Furthermore, cognitive improvement has been observed in patients undergoing long-term melatonin treatment (Wade et al., 2014). Another recommendation to improve sleep time is exercise. People with AD who perform regular physical activity are known to have fewer sleep-related problems when compared to patients who do not exercise regularly (Christofoletti et al., 2011). Exposure to light in the morning is also one of the strategies that, combined with exercise, increases sleep time, and decreases awakenings at night in AD patients (Memon et al., 2020). Girls between the ages of 9 to 11 years that sleep for enough hours for their age but bear social jetlag have a higher BMI. This denotes the importance of the circadian component of sleep and its relationship to metabolic problems that can occur before adulthood. Another important factor to consider is diet. Older adults under a Mediterranean diet which is high in vegetables, cereals, fruits, and seeds have a better quality of sleep (Mamalaki et al., 2018). The extension of sleep time has been proposed as an alternative to reduce metabolic problems and the risk to develop obesity (Hoddy et al., 2020). In other studies, regarding sleep extension, increasing participants’ bedtime has been found to reduce their intake of foods high in fat and carbohydrates (Al Khatib et al., 2018).

It is imperative to recognize the importance of sleep as an essential process for physiology and cognitive function, as well as to acknowledge that the cost of each night with insufficient sleep goes beyond poor concentration and bad mood, it becomes an important risk factor to develop serious conditions such as insulin resistance that, eventually, could make us even more susceptible to neurodegenerative diseases (Figure 2).

## Author Contributions

JG-A: conceptualization, investigation, writing, review and editing, and figures designing. RM-H: investigation, writing, review and editing, validation, and language editing. MG-R and NG-V: writing, supervision, review and editing, and validation. MC and JV-M: supervision, review and editing, and validation. GH-A: conceptualization, supervision, writing, review, editing, and validation. All authors contributed to the article and approved the submitted version.

## Conflict of Interest

The authors declare that the research was conducted in the absence of any commercial or financial relationships that could be construed as a potential conflict of interest.

## Publisher’s Note

All claims expressed in this article are solely those of the authors and do not necessarily represent those of their affiliated organizations, or those of the publisher, the editors and the reviewers. Any product that may be evaluated in this article, or claim that may be made by its manufacturer, is not guaranteed or endorsed by the publisher.
